# Chromosomal abnormalities detected by karyotyping among patients with secondary amenorrhea: a retrospective study

**DOI:** 10.1590/1516-3180.2022.0426.R1.14012023

**Published:** 2023-04-07

**Authors:** Marina da Rocha Besson, Mateus dos Santos Taiarol, Eliaquim Beck Fernandes, Isadora Bueloni Ghiorzi, Maurício Rouvel Nunes, Paulo Ricardo Gazzola Zen, Rafael Fabiano Machado Rosa

**Affiliations:** I BSc. Master´s Student, Postgraduate Program in Pathology, Universidade Federal de Ciências da Saúde de Porto Alegre (UFCSPA), Porto Alegre (RS), Brazil.; II Undergraduate Student, Department of Clinical Medicine, Universidade Federal de Ciências da Saúde de Porto Alegre (UFCSPA), Porto Alegre (RS), Brazil.; III Undergraduate Student, Department of Clinical Medicine, Universidade Federal de Ciências da Saúde de Porto Alegre (UFCSPA), Porto Alegre (RS), Brazil.; IV Undergraduate Student, Department of Clinical Medicine, Universidade Federal de Ciências da Saúde de Porto Alegre (UFCSPA), Porto Alegre (RS), Brazil.; V BSc. Doctoral Student, Postgraduate Program in Pathology, Universidade Federal de Ciências da Saúde de Porto Alegre (UFCSPA), Porto Alegre (RS), Brazil.; VI PhD. Professor, Departments of Clinical Medicine and Clinical Genetics, Universidade Federal de Ciências da Saúde de Porto Alegre (UFCSPA), Porto Alegre (RS), Brazil.; VII PhD. Professor, Departments of Clinical Medicine and Clinical Genetics, Universidade Federal de Ciências da Saúde de Porto Alegre (UFCSPA), Porto Alegre (RS), Brazil.

**Keywords:** Amenorrhea, Menopause, premature, Primary ovarian insufficiency, Abnormal karyotype, Chromosomes, human, x, Secondary amenorrhea, Karyotype, Chromosomal abnormalities, Short stature

## Abstract

**BACKGROUND:**

Chromosomal abnormalities (CAs) have been described in patients with secondary amenorrhea (SA). However, studies on this association are scarce.

**OBJECTIVES:**

To evaluate the frequency and types of CAs detected by karyotyping in patients with SA.

**DESIGN AND SETTING:**

This retrospective study was performed in a reference clinical genetic service in South Brazil.

**METHODS:**

Data were obtained from the medical records of patients with SA who were evaluated between 1975 and 2022. Fisher’s bicaudate exact test and Student’s t-test were used, and P < 0.05 was considered significant.

**RESULTS:**

Among 43 patients with SA, 14 (32.6%) had CAs, namely del (Xq) (n = 3), 45,X (n = 2), 46,X,r(X)/45,X (n = 2), 46,XX/45,X (n = 1), 46,X,i(q10)/45,X (n = 1), 47,XXX (n = 1), 46,XX/47,XXX (n = 1), 46,XX/47,XX,+mar (n = 1), 45,XX,trob(13;14)(q10;q10)/46,XXX,trob(13;14)(q10;q10) (n = 1), and 46,XX,t(2;21)(q23;q11.2) (n = 1). Additional findings were observed mostly among patients with CA compared with those without CA (P = 0.0021). No difference in the mean age was observed between the patients with SA with or without CAs (P = 0.268025).

**CONCLUSIONS:**

CAs are common among patients with SA, especially those with short stature and additional findings. They are predominantly structural, involve the X chromosome in a mosaic, and are compatible with the Turner syndrome. Patients with SA, even if isolated, may have CAs, particularly del (Xq) and triple X.

## INTRODUCTION

Amenorrhea is a symptom, not a proper condition, characterized by an alteration in the menstrual cycle that affects 2%–5% of women of childbearing age.^1,2^ Secondary amenorrhea (SA) corresponds to most amenorrhea cases and affects 3%–4% of women of childbearing age. It is defined by the cessation of menstruation for a minimum period of 3 months in patients with previously regular cycles or 6 consecutive months in women who have had at least one previous menstruation.^1,3^ The diagnostic evaluation of patients with SA begins with assessing patient history, followed by conducting physical examination, laboratory tests, and imaging.^4,5^ Although karyotyping is not routinely performed, it can be an important test.

Considering hormones, SA can be classified as having a central (hypothalamic–hypophyseal) or peripheric (ovarian) origin.^1,6^ Hypothalamic disorders are some of the most common causes of amenorrhea, including SA.^7^ SA might also have a peripheric origin because of a primary ovarian insufficiency that occurs after menarche and before the age of 40 years.^8^

Several factors can be related to SA, including genetics, the environment, and the interactions between them. Among genetic causes, chromosome abnormalities (CAs),^1,6^ which are generally identified by cytogenetic tests (e.g. karyotyping) are observed. Most CAs are chromosome X-related, similar to the Turner syndrome (TS).^6,9^

Thus, determining the cause of SA is essential for the appropriate management and treatment of patients.^5^ However, studies on SA and CAs are few, and most of them are case reports or case series.^9–11^

## OBJECTIVE

This study aimed to evaluate the frequency and types of CAs detected by karyotyping in a sample of patients with SA, attempting to correlate these CAs with other clinical features observed in such patients.

## METHODS

This retrospective study was performed in the Department of Genetics in the southern region of Brazil. The sample comprised patients with SA who were examined between 1975 and 2022. Clinical data and information on karyotypes were gathered from the medical records and clinical protocols of the patients. All the patients underwent a GTG-banding karyotype test in the same laboratory, following the modified technique of Yunis.^12^

The variables present in the clinical protocol were age at first evaluation, medical specialty from which the patients were referred, family history of amenorrhea, age at menarche and cessation of menstruation, period between age of menarche and cessation of menstruation, anthropometric measurements, data of physical examination, presence or absence of other comorbidities, syndromic appearance, and results of hormone levels, imaging exams, and karyotype.

Anthropometric measures were evaluated according to the Growth Charts of the Centers for Disease Control and Prevention.^13^ The patients were also classified as either syndromic or non-nonsyndromic by a single clinical geneticist. Additionally, the patients were divided into those with hypogonadotropic hypogonadism or hypergonadotropic hypogonadism.^6^ The CAs identified were described according to the International System for Human Cytogenetic Nomenclature 2016.^14^ Furthermore, they were classified into numeric or structural and with or without mosaicism.

For data analysis, Fisher’s exact test (https://www.socscistatistics.com/tests/fisher/default2.aspx) and Student’s T-test (https://www.socscistatistics.com/tests/studentttest/default.aspx) were used to compare frequencies and means, respectively. Significance was set at P < 0.05. The study was approved by the Ethics Committees of the Federal University of Health Sciences of Porto Alegre (UFCSPA) CAAE: 09909712.3.3001.5345 on January 12, 2018, and of Presidente Vargas Mother and Child Hospital (HMIPV) CAAE: 09909712.3.1001.5329 on October 10, 2017.

## RESULTS

The sample comprised 43 patients, with age at first evaluation ranging from 17 to 47 years (mean, 28.8). Most of the patients were referred from the Department of Gynecology (61%), followed by the Department of Endocrinology (36%) and the Department of Neurology (3%). The age at menarche was 9–18 years (mean, 13.1), and the age at menstruation cessation was 11–34 years (mean, 21.5). The period between menarche and cessation of menstruation was 1–21 years (mean, 8.2).

A family history of amenorrhea was noted in 17.2% of the patients. Regarding physical appearance, 10 patients (23.3%) had additional clinical features other than SA, and 9.3% were considered syndromic. The main findings were short stature (17.6%), intellectual deficit (9.3%), hypothyroidism (5.6%), congenital heart disease (3.7%), and hearing loss (2.8%). Regarding hormonal profiles, 93.3% of the patients had hypergonadotropic hypogonadism, whereas 6.7% had hypogonadotropic hypogonadism.

CAs were identified in 14 patients (32.6%), and the number of analyzed cells was 15–100 (average, 38.7). Structural anomalies involving the X chromosome (64.3%) were the predominant CAs (Table 1). Numeric CAs were observed in 35.7% of the patients, and mosaicism was noted in seven patients (50%). Six patients (14%) were diagnosed with TS, and 3 (7%) had triple X syndrome.

**Table 1.** Chromosomal findings observed among the patients with secondary amenorrhea

Karyotypic findings	Number of patients (%)	Patients with syndromic aspect (%)	Patients with additional findings (%)
Normal (46.XX)	29 (67.4)	2 (50)	3 (30)
Abnormal	14 (32.6)	2 (50)	7 (70)
46,X,del(Xq)	3 (7)	–	–
q13q26	1 (2.3)	–	–
q22q28	1 (2.3)	–	–
q24q28	1 (2.3)	–	–
45,X	2 (4.7)	–	2 (20)
mos 45,X/46,X,r(X)	2 (4.7)	–	2 (20)
mos 45,X[28]/46,X,r(X)[7]	1 (2.3)	–	1 (10)
mos 45,X[36]/46,X,r(X)[6]	1 (2.3)	–	1 (10)
mos 45,X/46,XX	1 (2.3)	–	–
mos 45,X[3]/46,XX[50]	1 (2.3)	–	–
mos 45,X/46,X,i(q10)	1 (2.3)	–	1 (10)
mos 45,X[2]/46,X,i(q10)[48]	1 (2.3)	–	1 (10)
47,XXX	1 (2.3)	–	–
mos 47,XXX/46,XX	1 (2.3)	–	–
mos 47,XXX[95]/46,XX[2]	1(2.3)	–	–
mos 46,XXX,der(13;14)(q10;q10)/45,XX,der(13;14)(q10;q10)	1 (2.3)	1 (25)	1 (10)
mos 46,XXX,der(13;14)(q10;q10)[15]/45,XX,der(13;14)(q10;q10)[72]	1 (2.3)	1 (25)	1 (10)
mos 47,XX,+mar/46,XX	1 (2.3)	–	–
mos 47,XX,+mar[37]/46,XX[4]	1 (2.3)	–	–
46,XX,t(2;21)(q23;q11.2)	1 (2.3)	1 (25)	1 (10)
**Total**	**43 (100)**	**4 (100)**	**10 (100)**

When comparing the groups with and without CAs, those with CAs only had more additional findings (P = 0.0021). We did not identify significant differences in mean age at the first evaluation (P = 0.9612), mean age of SA (P = 0.2680), periods between age at menarche and cessation of menstruation (P = 0.4285), hormonal profile (P = 1.0000), or syndromic aspect (P = 0.5855) (Table 2) between the two groups. The mean age of SA in patients with TS was 17.2 years old (range, 15–24 years), and the normal karyotype was 22.5 (ranging, 11–34 years). No significant difference in the mean values was observed between the two groups (P = 0.1617). The frequency of CAs was 30.7% among the patients with hypergonadotropic hypogonadism and 33.3% among those with hypogonadotropic hypogonadism.

**Table 2.** Clinical and laboratory findings verified among the patients with normal karyotype and chromosomal abnormalities

Clinical features	Normal karyotype	Chromosomal abnormalities	P
**Age at first evaluation (years)**	28.8 (17–47)	28.9 (19–42)	0.9612
**Mean age of secondary amenorrhea (years)**	22.5 (11–34)	19.7 (12–18)	0.2680
**Period between age of menarche and cessation of menstruation (years)**	9 (1–21)	6.9 (1–15)	0.4285
**Hormonal profile**			
Hypergonadotropic hypogonadism (%)	27 (93,1)	13 (93)	1.0000
Hypogonadotropic hypogonadism (%)	2 (6.9)	1 (7)	
**Syndromic appearance (%**)	2 (6.9)	2 (14.3)	0.5855
**Additional findings (%)**	2 (6.9)	7 (50)	0.0021
**Total**	**29 (67.4)**	**14 (32.6)**	

## DISCUSSION

SA may have multiple causes. Genetic causes include single gene alterations and CAs.^1,6^ CAs have been associated with SA in 3.8% to 44% of the cases; this variation occurs most likely because of the different forms of selection of individuals in each study, as well as the varying sample sizes.^6^ In our study, a frequency of 32.6% was recorded. This elevated index may be related to the place where the patients were evaluated, which was the Department of Clinical Genetics (patients with SA were commonly evaluated in other departments, especially in the Department of Gynecology and Endocrinology, from which almost all the patients in our sample were referred from). Therefore, they were selected before the clinical genetics evaluation.

CAs described in association with SA can be numeric or structural, and they can occur in an isolated form or involve more than one cellular lineage.^6,9^ Furthermore, these patients generally exhibit a wide range of phenotypic abnormalities, other clinical features, and hormonal profiles.^15^

In the literature, CAs associated with SA have been described to mainly affect the X chromosome,^9^ a finding that accords with that of our study reporting that among patients with CAs, 85.7% had abnormalities involving the X chromosome. In our sample, the main alteration involving the X chromosome was the deletion of parts of its long arm, which corresponded to 21.4% of the CA cases. Proper functioning of the gonads depends on the integrity of both X chromosomes.^9^ A region of great importance related to normal ovary development and functioning is localized at the long arm of the X chromosome and ranging from Xq13.3 to Xq27.^16^ Thus, deletions involving the long arm of this chromosome, as observed in three of our patients, can result in ovarian failure. Thus, considering that these patients often have primary amenorrhea or SA but without a short stature or other features of TS,^17^ as observed in our patients with Xq deletions, is crucial.

Additionally, loci located in the long arm of the X chromosome (named POF1 and POF2) are associated with premature ovarian failure and infertility. Locus POF1 involves the segment between Xq26 to Xqter, while locus POF2 involves the segment between Xq13.3 to Xq22.^10,11,16^ Locus POF1 and locus POF2 are clinically associated with ovarian failure in patients aged 24–29 years old and in those aged 16–21 years old, respectively.^18^ In our sample, the two patients presenting deletions involving POF1 (regions q22q28 and q24q28) were diagnosed with ovarian failure at 27 and 28 years old, respectively. Meanwhile, the patient with the deletion involving the POF2 (region q13q26) was diagnosed with ovarian failure at 15 years old. As previously mentioned, these findings are consistent with those in the literature.^11,18^

The genes placed in the POF1 and POF2 loci of X chromosome are as follows: *CHM* (Xq21.1) (OMIM *300390), *POF1B* (Xq21.1) (OMIM *300603), *DACH2* (Xq21.3) (OMIM *300608), *DIAPH2* (Xq22) (OMIM *300108), *NXF5* (Xq22.1) (OMIM *300319), *COL4A6* (Xq22.3) (OMIM *303631), *PGRMC1* (Xq24) (OMIM *300435), *XPNPEP2* (Xq25) (OMIM *300145), *FMR1* (Xq27.3) (OMIM *309550), and *FMR2* (Xq28) (OMIM *300806).^19, 20, 21, 22, 23, 24, 25, 26^ However, despite the description of all these candidate genes, the cause of premature ovarian failure remains unknown in most cases.^27,28^

In our study, 14% of the patients had TS, a condition characterized by total or partial absence of the X chromosome. As observed in our sample, it can present as different chromosomal constitutions.^17^ It occurs in approximately 1 in 2,500–5,000 women and is commonly diagnosed later, on average at the age of 15 years.^29^ Patients with this syndrome may have typical clinical characteristics that include cardiac, skeletal, and endocrine abnormalities, including hypothyroidism and short stature.^17,30^ However, this clinical spectrum ranges from a typical appearance to a presentation without clinical characteristics or minimal findings.^30^ These characteristics and clinical variability were also observed in our sample, in which the most consistent finding was short stature, which was present in all the patients. Moreover, of the patients with short stature in our sample, 71.4% were diagnosed with TS, and this finding was associated with the presence of the syndrome. Thus, a short stature in TS is due to the haploinsufficiency of the *SHOX* gene, which is located on the short arm of the X chromosome.^31^

Post-pubertal patients with TS commonly present with hypergonadotropic hypogonadism due to ovarian dysgenesis that leads to premature ovarian failure. Therefore, most patients experience pubertal delay and primary amenorrhea. However, SA has also been observed, especially when associated with mosaicism.^17,30^ This was also observed in our sample, in which 66.7% of the patients with TS presented with mosaicism. Moreover, previous studies have demonstrated that one third of patients with TS present with spontaneous thelarche, which also occurs more often in patients with mosaicism.^32^ Regular menstrual cycles occur in approximately 6% of these patients.^33^ Hence, TS might be only diagnosed later in life.^17^ In our sample, the patients with TS were diagnosed at approximately 26.2 years old. Their low age of ovarian failure, ranging from 15 to 21 years (mean: 17.2 years), is an important finding to highlight.

As previously mentioned, the chromosome constitution of patients with TS is variable.^17^ with total monosomy of X (45,X) being the main alteration and representing 40%–50% of all cases.^17,29^ These patients generally have more phenotypic abnormalities, such as a short stature^34^ and premature ovarian failure leading to primary amenorrhea or SA^35^ in which the streak ovaries commonly lack follicles. However, the clinical spectrum can be variable.^17,29^ In our sample, 2 of the 6 patients with TS had a 45,X constitution. Short stature was the only additional finding in both patients, and cessation of menstrual cycles occurred at 15 and 16 years of age in the two patients.

TS may also be caused by short-arm monosomy of the X chromosome.^17^ In our sample, 3 of 6 patients with TS had this chromosome particularity. Two of them had the ring form of the X chromosome [r(X)] and mosaicism (with an associated 45,X lineage). The ring form of the X chromosome occurs because of the deletion of parts of its short and long arms, along with their posterior fusion. This constitution is related to atypical and severe cases of TS, including intellectual deficiency. This may occur because of *XIST* changes, which are the main genes responsible for controlling X chromosome inactivation. Therefore, modifications involving this region cause a greater expression of chromosomal material, leading to a higher frequency of abnormalities, including atypical abnormalities, such as microcephaly, agenesis of the corpus callosum, and seizures. The size of the ring X chromosome lacking *XIST* and, therefore, unable to become inactivated, correlates with phenotype severity in some cases. By contrast, patients with large rings that undergo selective X-chromosome inactivation are frequently associated with a more normal phenotype.^36^

Most patients with ring X chromosomes are infertile, as are other patients with TS. The gonads comprise striae with no follicular development. However, some patients are fertile and may transmit the ring X-chromosome to their progeny. In these rare cases, the ring is commonly large, with breakpoints on the short arm at bands p13 and p22. On the long arm, the breakpoints are at band q24 or q27.^36^ In our study, patients with ring X chromosome did not present atypical clinical features (possibly due to mosaicism with the associated 45,X lineage). One patient presented with hypothyroidism; however, as mentioned previously, this is a common finding in TS. The age of menstrual cycle cessation was low, as observed in patients with a 45,X constitution, and ranged from 15 to 16 years. This premature age of SA might be influenced by the 45,X lineage present in association with the ring X chromosome lineage that was observed in both patients.

Approximately 20%–30% of patients with TS are carriers of an isochromosome of the X chromosome long arm.^17^ This finding was observed in one patient in our sample in association with a 45,X lineage. Patients with isochromosomes commonly present with clinical features similar to those of patients with a 45,X constitution. However, they have a higher frequency of dysgenetic gonads, primary amenorrhea, SA, short stature, and major anomalies, such as congenital heart defects and renal malformations. Autoimmune diseases, such as Hashimoto thyroiditis, are also common in these cases.^37^ The patient with an X long arm isochromosome in our sample had a short stature without any associated malformations or autoimmune diseases. Her menstrual cycle was interrupted at 24 years of age, which is an age older than that commonly described among patients with TS.

As previously mentioned, TS may occur in a mosaicistic constitution. The most common constitution is associated with a normal cellular lineage,^17^ as detected in one patient in our sample. This constitution is observed in 15% to 25% of TS cases.^17^ These patients commonly present with a higher stature, a lower frequency of major abnormalities, and, most commonly, SA, when compared to those with a 45,X karyotype. Menarche has been described in approximately 2%–5% of cases. These findings may be attributable to the presence of a normal cell lineage.^9^

Triple X syndrome is characterized by an extra X chromosome resulting from the nondisjunction of sexual chromosomes during the first meiotic division, and it occurs in 1 in every 1,000 women. This alteration is highly related to advanced maternal age. The phenotype observed in each individual with triple X might vary, and only approximately 10% of the cases are diagnosed.^38^ Although sexual development and ovarian function are normal in most of these patients, ovarian dysfunction may manifest as premature menopause, SA, or oligomenorrhea.^13,39^ The first report of triple X syndrome involving a 35-year-old woman with SA was by Jacobs et al.^40^ In our study, one patient had triple X syndrome and hypergonadotropic hypogonadism due to premature ovarian failure. SA was her only finding, and her menstrual cycle was interrupted at 19 years of age.

Although more rarely (10% of cases), triple X might also occur in mosaicism.^41^ Temoçin et al.^42^ have reported that 1 in every 9 patients (11.1%) with SA has 46,XX/47,XXX mosaicism. Additionally, a study by Ayed et al.^43^ has revealed that in a sample of 40 patients with SA due to premature ovarian failure, 5 patients (12.5%) had CAs, and 2 (40%) had triple X in mosaic (1 with 45,X/47,XXX constitution and 1 with 46,XX/47,XXX constitution; 5% of all the patients had premature ovarian failure. In our sample, 2 patients with triple X mosaicism were identified. The first was mosaicism with a normal lineage. The patient was not syndromic and had premature ovarian failure. Her menstrual cycle was interrupted at 19 years of age. Interestingly, 1 patient with triple X mosaicism had a balanced Robertsonian translocation between chromosomes 13 and 14. Clinically, she had an important intellectual deficit, behavioral disorder (with aggressive episodes), short stature, obesity, and hypothyroidism, in addition to SA due to hypogonadotropic hypogonadism. In this case, the occurrence of triple X syndrome related to maternal uniparental disomy of chromosome 14 due to the translocation between chromosomes 13 and 14 in both lineages cannot be disregarded. Interestingly, Bertini et al.^44^ have described a patient with similar clinical findings and uniparental maternal disomy of chromosome 14, which was also associated with Robertsonian translocation between chromosomes 13 and 14.

Chromosome markers comprise a few structurally abnormal chromosomes of unknown origin. One patient with mosaicism involving one normal cellular lineage with a supernumerary marker chromosome (47,XX+mar) was observed in our sample. In these cases, the use of molecular cytogenetic techniques, such as fluorescent *in situ* hybridization, is recommended to determine the marker chromosome origin.^17^ However, our patient did not undergo this test since she was evaluated 20 years ago. As for her clinical condition, she did not have a syndromic appearance, and SA was the only finding.

Interestingly, one patient in our sample had an apparently balanced *de novo* translocation involving chromosomes 2 and 21 with normal parental karyotypes. The patient had an intellectual deficit associated with SA. First, no genes associated with her clinical condition were located at translocation breakpoints in the literature. Additionally, at the time of evaluation, molecular cytogenetic techniques were still unavailable.

The frequency of mosaicism identified in our study (50%) and that described in the literature (40%–100%) are both high.^6,42,43,45^ It should also be recalled that most patients with TS presenting with SA have a mosaic constitution.^17,30^ Hence, among the cases of SA, the number of analyzed cells in karyotypic evaluation should be higher, targeting the detection of potential mosaicism (this is in agreement with the higher cell count that is routinely performed in mosaicism suspicion, that is, in general, 100).^46^

## CONCLUSION

As CAs are common among patients with SA, cytogenetic analysis by karyotyping is important, especially in patients with short stature and additional findings. The main types of CAs observed were structural, involving the X chromosome, and compatible with a TS diagnosis. Several CAs occurred in the mosaic. This finding is also commonly reported in previous studies, which have suggested that patients with SA should be evaluated by karyotyping with more cells counted as a precaution.

However, the absence of additional findings other than SA did not exclude karyotype indication. This is because a significant number of patients, even those with CAs, may have SA as an isolated finding, especially in patients with the Xq deletion and triple X syndrome (with or without mosaicism). In our sample, we did not observe a difference in SA age between patients with and without CAs. However, we cannot rule out the influence of the small sample size on this result.

Therefore, the diagnosis of CAs, especially when performed in the early stages, is possible and important for better management of patients with SA.

## References

[1] Rajangam S, Nanjappa L (2007). Cytogenetic studies in amenorrhea. Saudi Med J..

[2] Dutta UR, Ponnala R, Pidugu VK, Dalal AB (2013). Chromosomal abnormalities in amenorrhea: a retrospective study
and review of 637 patients in South India. Arch Iran Med..

[3] Cortés-Gutiérrez EI, Dávila-Rodríguez MI, Vargas-Villarreal J, Cerda-Flores RM (2007). Prevalence of chromosomal aberrations in Mexican women with
primary amenorrhoea. Reprod Biomed Online..

[4] Klein DA, Poth MA (2013). Amenorrhea: an approach to diagnosis and
management. Am Fam Physician..

[5] Kwon SK, Chae HD, Lee KH (2014). Causes of amenorrhea in Korea: experience of a single large
center. Clin Exp Reprod Med..

[6] Safai A, Vasei M, Attaranzadeh A, Azad F, Tabibi N (2012). Chromosomal abnormality in patients with secondary
amenorrhea. Arch Iran Med..

[7] Deligeoroglou E, Athanasopoulos N, Tsimaris P (2010). Evaluation and management of adolescent
amenorrhea. Ann NY Acad Sci..

[8] Goswami D, Conway GS (2007). Premature ovarian failure. Horm Res..

[9] Rosa RF, Dibi RP, Picetti J dos S (2008). Amenorréia e anormalidades do cromossomo X [Amenorrhea and X
chromosome abnormalities]. Rev Bras Ginecol Obstet..

[10] Hassum PA, Silva IDC, Vereschi ITN (2001). O espectro das falências ovarianas ligadas ao cromossomo
X. Arq Bras Endocrinol Metab..

[11] Badalotti M, Arent A, Polanczick A, Petracco R, Petracco A (2006). Falência ovariana precoce associada a deleção no braço longo do
cromossomo: relato de dois casos e revisão da literatura. Rev Bras Ginecol Obstet..

[12] Yunis JJ (1981). New chromosome techniques in the study of human
neoplasia. Hum Pathol..

[13] Jones KL (2006). Smith’s recognizable patterns of human malformation.

[14] McGowan-Jordan J, Simons A, Schmid M., ISCN 2016 (2016). An International System for Human Cytogenetic Nomenclature.

[15] Ghosh S, Roy S, Halder A (2020). Study of frequency and types of chromosomal abnormalities in
phenotypically female patients with amenorrhea in Eastern Indian
population. J Obstet Gynaecol Res..

[16] Persani L, Rossetti R, Cacciatore C, Bonomi M (2009). Primary ovarian insufficiency: X chromosome defects and
autoimmunity. J Autoimmun..

[17] Gravholt CH, Andersen NH, Conway GS (2017). International Turner Syndrome Consensus Group. Clinical practice
guidelines for the care of girls and women with Turner syndrome: proceedings
from the 2016 Cincinnati International Turner Syndrome
Meeting. Eur J Endocrinol..

[18] McAuley K, Cambridge L, Galloway S, Sullivan J, Manning P (2000). De novo deletion of Xq associated with premature ovarian
failure. Aust N Z J Med..

[19] Bione S, Sala C, Manzini C (1998). A human homologue of the *Drosophila melanogaster*
diaphanous gene is disrupted in a patient with premature ovarian failure:
evidence for conserved function in oogenesis and implications for human
sterility. Am J Hum Genet..

[20] Lorda-Sanchez IJ, Ibañez AJ, Sanz RJ (2000). Choroideremia, sensorineural deafness, and primary ovarian
failure in a woman with a balanced X-4 translocation. Ophthalmic Genet..

[21] Prueitt RL, Chen H, Barnes RI, Zinn AR (2002). Most X;autosome translocations associated with premature ovarian
failure do not interrupt X-linked genes. Cytogenet Genome Res..

[22] Bione S, Rizzolio F, Sala C (2004). Mutation analysis of two candidate genes for premature ovarian
failure, DACH2 and POF1B. Hum Reprod..

[23] Mansouri MR, Schuster J, Badhai J, Stattin El, Losel R (2008). Alterations in the expression, structure and function of
progesterone receptor membrane component-1 (PGRMC1) in premature ovarian
failure. Hum Mol Genet..

[24] Bertini V, Ghirri P, Bicocchi MP, Simi P, Valetto A (2010). Molecular cytogenetic definition of a translocation t(X;15)
associated with premature ovarian failure. Fertil Steril..

[25] Nishimura-Tadaki A, Wada T, Bano G (2011). Breakpoint determination of X;autosome balanced translocations in
four patients with premature ovarian failure. J Hum Genet..

[26] Dixit H, Rao L, Padmalatha V (2010). Genes governing premature ovarian failure. Reprod Biomed Online..

[27] Goswami D, Conway GS (2005). Premature ovarian failure. Hum Reprod Update..

[28] Shelling AN (2010). Premature ovarian failure. Reproduction..

[29] Gravholt CH, Viuff MH, Brun S, Stochholm K, Andersen NH (2019). Turner syndrome: mechanisms and management. Nat Rev Endocrinol..

[30] Davenport ML (2010). Approach to the patient with Turner syndrome. J Clin Endocrinol Metab..

[31] Martins RRS, Ramos HIB, Llerena JC, Almeida JCC (2003). Investigação clínica e genética em meninas com baixa estatura
idiopática. Arq Bras Endocrinol Metab..

[32] Tanaka T, Igarashi Y, Ozono K (2015). Frequencies of spontaneous breast development and spontaneous
menarche in Turner syndrome in Japan. Clin Pediatr Endocrinol..

[33] Pasquino AM, Passeri F, Pucarelli I, Segni M, Municchi G (1997). Spontaneous pubertal development in Turner’s syndrome. Italian
Study Group for Turner’s Syndrome. J Clin Endocrinol Metab..

[34] Jacobs P, Dalton P, James R (1997). Turner syndrome: a cytogenetic and molecular
study. Ann Hum Genet..

[35] Ogata T, Matsuo N (1995). Turner syndrome and female sex chromosome aberrations: deduction
of the principal factors involved in the development of clinical
features. Hum Genet..

[36] Leppig KA, Disteche CM (2001). Ring X and other structural X chromosome abnormalities: X
inactivation and phenotype. Semin Reprod Med..

[37] Roy S, Halder A, Pal P, Dutta A, Ghosh S (2015). Isochromosome Xq: not a rare finding in short stature females
with amenorrhea. Int J Curr Res..

[38] Otter M, Schrander-Stumpel CT, Curfs LM (2010). Triple X syndrome: a review of the literature. Eur J Hum Genet..

[39] Sugawara N, Maeda M, Manome T, Nagai R, Araki Y (2013). Patients with 47, XXX karyotype who experienced premature ovarian
failure (POF): two case reports. Reprod Med Biol..

[40] Jacobs PA, Baikie AG, Brown WM (1959). Evidence for the existence of the human “super
female”. Lancet..

[41] Pouresmaeili F, Fazeli Z (2014). Premature ovarian failure: a critical condition in the
reproductive potential with various genetic causes. Int J Fertil Steril..

[42] Temoçin K, Vardar MA, Süleymanova D (1997). Results of cytogenetic investigation in adolescent patients with
primary or secondary amenorrhea. J Pediatr Adolesc Gynecol..

[43] Ayed W, Amouri A, Hammami W (2014). Cytogenetic abnormalities in Tunisian women with premature
ovarian failure. C R Biol..

[44] Bertini V, Fogli A, Bruno R (2017). Maternal uniparental disomy 14 (Temple syndrome) as a result of a
Robertsonian translocation. Mol Syndromol..

[45] Opitz O, Zoll B, Hansmann I, Hinney B (1983). Cytogenic investigation of 103 patients with primary or secondary
amenorrhea. Hum Genet..

[46] Hook EB (1977). Exclusion of chromosomal mosaicism: tables of 90%, 95% and 99%
confidence limits and comments on use. Am J Hum Genet..

